# Dual Ventricular Response or 1 : 2 Atrioventricular Conduction in Dual Atrioventricular Nodal Physiology

**Published:** 2008-04-01

**Authors:** Johnson Francis, MN Krishnan

**Affiliations:** Department of Cardiology, Calicut Medical College, Kerala, India

**Keywords:** Dual ventricular response, 1:2 atrioventricular conduction, dual atrioventricular nodal physiology

Dual ventricular response to a single supraventricular impulse is an interesting possibility in the  presence of dual atrioventricular nodal physiology. Double His bundle and ventricular responses to a single atrial impulse caused by a simultaneous fast and slow pathway conduction is the hallmark of this condition. One of the earliest descriptions of simultaneous conduction through both atrioventricular (AV) nodal pathways was by Csapo G [1], who described various electrocardiographic patterns due to simultaneous conduction through dual AV nodal pathways. Activation through triple AV nodal pathways has also been described [2,3]. In one case2 an atrial impulse evoked double ventricular response due to simultaneous activation of the slow and fast pathway. The next impulse activated the ventricles through the intermediate pathway, The net result was a narrow QRS tachycardia with irregular RR intervals. In another case [3] an incessant form of complex supraventricular tachycardia was noted, with simultaneous conduction over multiple AV nodal pathways. The tachycardia was successfully treated by ablation of intermediate and slow pathways. Over 20 cases non-reentrant supraventricular tachycardia with 1:2 AV conduction during sinus rhythm has been described in literature so far [4,19]

The major determinants of simultaneous anterograde fast and slow pathway conduction during sinus rhythm are: 1) Retrograde unidirectional block in both pathways 2) Critical conduction delay in the slow pathway and a long enough His-A interval to allow sequential conduction of impulse from both pathways [6]. The critical delay should be such that the impulse traveling through the slow pathway should reach the His bundle and  ventricles after the refractoriness following the fast pathway impulse (activation).

A mistaken diagnosis of atrial fibrillation may be entertained if the dual response is intermittent. Dixit S et al [17] found that 3 of the 456 consecutive patients referred for ablation of atrial fibrillation over a 3 year period had runs of dual response. The tachycardias were cured by slow pathway ablation. Hence they have suggested a stimulation protocol to identify such patients whose treatment is much simpler than AF ablation.

Non-reentrant supraventricular tachycardia due to 1:2 AV conduction has been described between 44 - 74 years of age and with duration of symptoms of up to 7 years [4-19]. It may be inducible with atrial and ventricular extrastimuli [4,8,19]. Slow pathway may have a longer refractory period than fast pathway [8]. Sometimes infusion of sympathomimetic agents is needed during atrial stimulation for inducing the tachycardia [19]. Treatment by slow pathway ablation has been successful in all cases in which it was reported [8,19]. One of the earlier reports noted disappearance of symptoms with flecainide [6].

Even tachycardiomyopathy secondary to non-reentrant atrioventricular nodal tachycardia has been described recently [18]. Treatment by slow pathway ablation resulted in full recovery of left ventricular function at 11 months of follow up.

In this issue of the journal Laszlo R et al [19] describes another case of non-reentrant supraventricular tachycardia due to 1:2 AV conduction, which was cured by slow pathway ablation.While this  mechanism is likely in this case, other possibilities must be considered. From the surface ECG,  alternating His bundle ectopics with  ventricular activation cannot be ruled out. This is more so as the EP study did not include a His bundle electrogram. Ideally an AH jump indicating blockage of the fast pathway conduction during sequential extrastimuli  should be demonstrated to establish the presence of a dual pathway. However, consistent reproduction of the tachycardia by atrial stimulaton  may indicate that alternating His ectopics are unlikely. This uncommon type of tachycardia has to be considered in the differential diagnosis of paroxysmal supraventricular tachycardia and irregular supraventricular rhythms mimicking atrial fibrillation.
